# Optimizing production of gluten‐free, sugar‐reduced cupcakes: Utilizing stevia as natural sweetener and psyllium as gluten substitute

**DOI:** 10.1111/1750-3841.70148

**Published:** 2025-03-27

**Authors:** Ezgi Kalkan, Medeni Maskan

**Affiliations:** ^1^ Engineering Faculty, Food Engineering Department Gaziantep University Gaziantep Turkey

**Keywords:** airfryer, cupcake, gluten‐free, psyllium, stevia

## Abstract

A comprehensive study was conducted to assess the effects of stevia (0%–1.2%), psyllium (0%–4%), and baking method (airfryer and oven) on the physical, textural, sensory, and pore characteristics of gluten‐free, sugar‐reduced cupcakes. The formulation was optimized using Response Surface Methodology‐Central Composite Face‐Centered Design (CCFD). Based on numerical optimization, the best cupcake formulation was predicted to contain 0.68% stevia and 1.82% psyllium, using the oven baking method. The optimized cupcake produced at this formulation exhibited the following characteristics: specific volume 38.75 mL/g, hardness 1710.85 gf, springiness 0.950, cohesiveness 0.638, gumminess 1119, chewiness 1042, resilience 0.350, and 44 pores. To analyze pore characteristics, the Otsu thresholding algorithm was employed, revealing a 2.5‐fold increase in the number of pores in the optimized cupcake compared to the control. This study demonstrates that the combination of stevia as natural sweetener and psyllium as gluten substitute significantly enhances the quality of a low‐calorie, gluten‐free cupcake. Furthermore, this formulation provides a promising, clean option for consumers with chronic diseases such as celiac and diabetes.

## 1 INTRODUCTION

Production of specific food products that maintain the same nutritional and textural quality is a critical requirement for individuals who suffer from chronic disorders such as celiac disease, gluten intolerance, or diabetes. Celiac disease impacts one in every hundred individuals. Celiac disease can only be handled with the lifelong restriction of all gluten‐containing foods from the diet (Zandonadi et al., 2009). However, not having enough gluten negatively impacts the structure, taste, and volume of baked goods (Filipčev et al., 2021). Gluten‐free baked goods are safe for celiac patients; however, gluten‐free diets are nutritionally deficient due to high carbohydrate content, low dietary fiber, and protein content, which does not meet customer expectations for healthy foods (Bascuñán et al., 2017).

In contrast to wheat flour, gluten‐free flour typically produces a cake with insufficient texture and lower volume. Numerous approaches have been used to improve the quality and texture of gluten‐free bakery items, including the use of hydrocolloids, enzymes, natural emulsifiers, and flour changes (Fratelli et al., 2021). Along with health benefits, plants high in dietary fiber have gained popularity since their functional features have opened up new opportunities in the food sector. As psyllium husk contains 85% water‐soluble fiber, it is responsible for psyllium's enhanced ability to hold water and gelling properties (E. A. N. Franco et al., 2020). Arabinoxylan is the most profound fiber, which makes up around 60% of the psyllium husk and comprises xylose and arabinose (Noguerol et al., 2022). Psyllium was the main gluten substitution in 16% of the specimens and 34% of the formulation in a study including 228 gluten‐free breads (Belorio & Gómez, 2021). The incorporation of psyllium into food products has been shown to improve the texture, appearance, and volume of the product, as well as the health of people who consume it in terms of glycemic control, cholesterol control, weight control, and the prevention of gastrointestinal disorders and constipation (E. A. N. Franco et al., 2020).

Cakes, with their flavor and soft texture, are the most alluring bakery items. However, it is classified as a high‐glycemic index product due to its high sugar content. Since overconsumption of sucrose can lead to health issues such as dental issues, weight gain, Type 2 diabetes, cardiovascular disease, and cancer, the food industry has concentrated on lowering sugar or substituting by natural sweeteners (Gao et al., 2019). Among more than 230 species, *Stevia rebaudiana* stands out as the only one that offers a sweet taste due to the presence of steviol glycosides, and it has long been used as a low‐calorie sweetener in South America, Mexico, Argentina, Asia, Japan, and Europe (Yadav et al., 2011). Stevia has specific glycosides of stevioside and rebaudioside A that are responsible for sweet taste but do not have calorie value (Sulaiman et al., 2022). According to the Expert Committee of FAO/WHO, JECFA, and FDA, stevia was approved as a sweetener, and the contribution of the food products is approved to be safe without side effects (Vatankhah et al., 2017). Numerous studies, driven for public health consideration, have highlighted stevia's anti‐diabetic, anti‐obesity, anti‐tumor, and anti‐microbial properties, as more than 60% of European consumers have started to search for sugarless products due to health concerns (Khilar et al., 2022). Also, stevioside and rebadioside A are non‐decay on teeth (Nikooie et al., 2015). A previous study identified stevia leaf extract as a safe alternative to sucrose because it contains no calories despite being 100–300 times sweeter than sucrose (Goyal et al., 2010). The complete or partial replacement of sucrose with stevia in baked products, including muffins, cakes, cookies, and bread, has been the subject of numerous studies (Gao et al., 2016). While stevia can be successfully involved into bakery products, the amount added must be mindfully monitored to avoid a bitter aftertaste, metallic flavor, and texture issues (Khilar et al., 2022).

The importance of selecting a baking procedure is critical for achieving the desired quality of food product with superior nutrition and energy efficiency (Mondal & Datta, 2008). Baking is defined as basically heating food in an oven with the aid of air, whereas frying is the dehydration of food through heat transfer in absence of direct contact with the food. During the baking process, heat is transferred through three primary modes: convection, radiation, and conduction. Among these, radiation is the most powerful, while convection is the least effective (Zareifard et al., 2009). Air frying is a prominent and developed method of cooking that involves circulating hot air around food (Ngan et al., 2023). The key distinction between an air fryer and a conventional oven lies in the modification of the heating chamber's shape to enhance air circulation (Ngan et al., 2023). The increased air velocity in an air fryer enhanced convective heat transfer, directly influencing the heat transfer mechanism, heat distribution, baking time, and the overall quality of the baked product (Zareifard et al., 2009). There is limited research in literature (Mior Zakuan Azmi et al., 2019; Shahapuzi et al., 2015a), and a thorough assessment of the effects of air fryer and oven baking methods on food product quality is essential.

Comparative studies on air fryer and oven methods regarding the effects of airflow on product's volume, texture, and sensory attributes are scarce. To the best of our knowledge, this study is the first to compare air frying and oven baking in the optimization of gluten‐free bakery products. In addition, although several studies have investigated the effects of psyllium as a gluten substitute, fat substitute, or functional ingredient in cakes and breads (Beikzadeh et al., 2016; Belorio & Gómez, 2021; Belorio et al., 2019) and the role of stevia as a sugar substitute in bakery products (Gökçe et al., 2023; Sanggramasari, 2019; Sulaiman et al., 2022), these investigations primarily focused on each component separately. Psyllium, a gluten substitute, and stevia, a natural sweetener, were combined for the first time to enhance the texture and sensory properties of gluten‐free baked goods in this study. In light of our preliminary trials, the greatest possible amount of sugar reduction without adversely affecting the structure and sweetness of the cake was discovered to be 60%. The following things were the novelty and the main focus of this study: (1) to determine the influence of stevia (Rebaudioside A), psyllium, and baking methods (air fryer and oven) on baking loss, specific volume, pore characteristics, textural (hardness, springiness, cohesiveness, gumminess, chewiness, resilience), and sensorial properties (appearance, flavor, texture, taste, sweetness, overall acceptability) (2) to optimize the parameters in the cake formulation using Response Surface Methodology (RSM) for the excellent cupcake preparation.

## 2 MATERIALS AND METHODS

### 2.1 Source of materials

The cake batter was made with gluten‐free flour mixture (corn starch, rice flour, pectin, xanthan gum, sodium bicarbonate, sodium acid pyrophosphate) (Sinangil), granulated sugar (Torku), fresh whole egg, sunflower oil (Yudum), milk (Sütaş), baking powder (Kenton®, sodium acid pyrophosphate, sodium bicarbonate, and corn starch), vanilla, and salt purchased from a local market in Gaziantep. For gluten measurement, a gluten analysis report was obtained from the manufacturer of gluten‐free flour (Sinangil). The report stated “Not detected” as the employed method allows for the precise determination and the control of gluten levels in products with a sensitivity of 5 mg/kg. It is stated that gluten level was below 5 mg/kg and not detected in the gluten‐free flour of the current research by the enzyme‐linked immunosorbent assay method. This method is approved by AOAC Official Method 2012.01 and AACC International and was conducted using the RIDASCREEN Gliadin test kit (R7001, R‐Biopharm). Psyllium husk powder (GuzelPharma) as a gluten alternative and stevia (Cargill, Rebaudiosit A component 95%) as a sugar substitute were also acquired.

### 2.2 Preparation of cake batter and baking

The control cake batter was formulated following the modified procedure outlined by Gao et al. (2016), which involved a combination of 100 g of flour, 100 g of sugar, 75 g of milk, 58 g of whole egg, 46 g of oil, 50 g of water, 2 g of vanilla, 2 g of baking powder, and 0.15 g of salt. Before the current experiment, a preliminary baking trial was conducted to ascertain the greatest extent of sugar reduction that would not adversely impact cake structure or sweetness. The sugar content of the modified cake was lowered by 60%. The experimental cake formulation and the cake batter used as controls are detailed in Table 1. These formulations are generated by Central Composite Face‐Centered Design (CCFD). CCFD created nine different cake formulations, including 0% stevia and 0% psyllium, which is also presented by control. Initially, eggs and sugar were whipped in a mixer (Braun, MQ7045X) at maximum speed for 2 min until a white cream was obtained to prepare the cake. The oil, milk, and water were then added into the cream and mixed for 1 min. To achieve a homogeneous structure, the dry ingredients (flour, baking powder, vanilla, and salt) were mixed in a separate cup before being incorporated with the creamy mixture and beaten for 2 min at the lowest speed. The cake batter was dispensed into cups with dimensions of 9 cm (top diameter) × 4 cm (bottom diameter) × 5 cm (height). The interior surfaces of the cups were coated with oil. Following that, the cups were baked by two different techniques: in an oven and an air fryer for 13 min at 175°C separately. Every cup held 23 g of cake batter. The oven (Arçelik 9542 CDG, 950 W) and air fryer (Philips HD9252/90, 1400 W) were preheated for 20 min each. After an hour of ambient temperature cooling, the air fryer and oven‐baked cupcakes were weighed. To make experimental cupcakes, the level of psyllium between 0% and 4% was added during the dry ingredient mixing phase, and the level of stevia between 0% and 1.2% was added as a sugar substitute by flour weight at the same time as sugar.

**TABLE 1:** Weight (g) of control cake and experimental cake formulations generated by Central Composite Face‐Centered Design (CCFD).

		Cake formulations							
Components	Control cake	0.6S0P	0.6S2P	0.6S4P	1.2S0P	1.2S2P	1.2S4P	0S2P	0S4P
Gluten free flour	100	99.40	97.40	95.40	98.80	96.80	94.80	98	96
Psyllium	0	0	2	4	0	2	4	2	4
Stevia	0	0.6	0.6	0.6	1.2	1.2	1.2	0	0
Sugar	40	40	40	40	40	40	40	40	40
Whole egg	58	58	58	58	58	58	58	58	58
Oil	46	46	46	46	46	46	46	46	46
Milk	75	75	75	75	75	75	75	75	75
Water	50	50	50	50	50	50	50	50	50
Salt	0.15	0.15	0.15	0.15	0.15	0.15	0.15	0.15	0.15
Baking powder	2	2	2	2	2	2	2	2	2
Vanilla	2	2	2	2	2	2	2	2	2

### 2.3 Determination of moisture content

The moisture level of gluten‐free cakes produced by optimal formulation with both baking types, as well as the control cake, was determined as it was of critical importance for baking loss. Moisture contents of cake batters were also determined, both control and optimized. The infrared moisture analyzer (Mettler Toledo MJ33) autonomously determined the moisture contents of the cakes and batters, which were then displayed as a percentage (Tugnolo et al., 2021).

### 2.4 Determination of baking loss

As stated by Liu et al. (2020), the baking loss of cupcakes was measured after thermal processing based on the percentage loss in weight of the cupcake batter to cake 1 h after baking. The baking loss of the gluten‐free cupcakes was measured using Equation (1).

(1) $$\mathrm{Baking}\;\mathrm{loss}\left(\%\right)=\frac{W_{\mathrm{batter}}-W_{\mathrm{cake}}}{W_{\mathrm{batter}}}\times 100$$

### 2.5 Determination of specific volume

The cakes' specific volume was determined using the grain displacement method applied by Ávila et al. (2017). A sample of the cupcake was weighed by a digital balance and recorded in grams. After that, rice was poured into a glass beaker that held 600 mL until it was full. A ruler is employed to scratch the container's surface to achieve symmetry in the grain surface. After the cupcake is positioned, it is subsequently filled with rice. The leftover rice was placed into a graduated cylinder, and the volume was expressed in mL. The specific volume of the cupcake was calculated by determining the ratio of the cake's volume to its weight, expressed in mL per gram.

### 2.6 Determination of texture properties

The texture profile analysis of cupcake samples in terms of hardness, springiness, cohesiveness, gumminess, chewiness, and resilience was assessed using a TPA/TA, XTplus Texture Analyzer (Stable Micro Systems). The analysis was performed using a trigger load of 5 g. The overnight after it was produced, the whole cupcakes were compressed by 50% using the P/36R probe (36 mm Dia Aluminum Radiused AACC, Stable Micro Systems) at a test speed of 2.00 mm/s. There was a 5‐s interval between the first and second compressions. The hardness in gram force was defined as the greatest value attained during the initial compression, whereas the cohesiveness was acquired as the ratio of the second compression area to the first compression. Chewiness was calculated by multiplying the gumminess and springiness values. The resilience was the height value linked with the cake's return after being subjected to pressure. Springiness was defined as the ratio of the distance between the highest forces between the second and first compression. The analyses were replicated three times, and the mean value was calculated (Ngan et al., 2023).

### 2.7 Pore characteristics

The image acquisition of gluten‐free, sugar‐reduced cupcakes was performed using the method performed by Amani et al. (2021). First, baked cupcakes were cut in cross‐section, and photos were captured at a fixed distance of 10 cm. A lamp that was 10 cm distant and nearly 30° from the surface was used for lighting up the samples.

Pore characteristics of the cupcakes regarding the number of pores were measured by using image analysis, utilizing the modified approaches from Amani et al. (2022). To begin, a cross‐sectional area of 12.5 mm × 41.66 mm was cropped from the center of the horizontally cut baked cupcakes, which was the photos' largest possible rectangular cross‐section. Subsequently, the images were transmitted to Fiji ImageJ, which is a software for analyzing image processing. The brightness/contrast function in ImageJ enhanced the quality of the cropped image. The images were processed to 8‐bit grayscale and then converted to a binary image using the Otsu thresholding algorithm. This is one of the most popular strategies for achieving appropriate thresholding. Pores were identified with an area greater than 0.38 mm^2^ and appeared as white areas in the binary pictures.

### 2.8 Sensory evaluation of cupcakes

Due to the plenty of samples, sensory analysis of only three cupcake samples (produced in oven at optimum conditions, produced in air fryer at optimum conditions, and control) was conducted by 23 panelists, 12 male, and 11 female, ranging in age from 25 to 55, who were mostly Gaziantep University students and employees. The Ethics Committee of Gaziantep University in Turkey assessed and approved the research (protocol number 012, record date: September 17, 2024). As a sample, one in four of each cake was randomly coded with three‐digit numbers, placed on a white plate, and served with water in between testing. The 7‐hedonic scale, which ranges from 1 (extremely disliked) to 7 (extremely liked), was used to assess appearance, flavor, texture, taste, sweetness, and overall acceptability. Panelists also evaluated the purchase intention test, which ranged from 1 (would definitely not buy) to 7 (would definitely buy), which showed how much they were willing to purchase. Acceptance testing is also called degree of liking. Consumers were presented with products and asked to indicate their degree of liking on a scale.

To determine the acceptance index (AI), Equation (2) was employed (Ávila et al. (2017).

(2) $$\mathrm{AI}\;=\frac{A}{B}\times 100$$

where *A* represents the average score gained by the product, and *B* represents the maximum score provided to the product. A satisfactory AI requires a decision criterion of at least 70%.

### 2.9 Experimental design, statistical analysis, and optimization of variables

In this study, CCFD with three independent variables was utilized for the optimization of gluten‐free, sugar‐reduced cupcake formulation. The independent variables were stevia (0%–1.2%), psyllium (0%–4%), and baking type (air fryer and oven). Stevia was used as a sugar substitute, and psyllium was added as a gluten substitute in order to strengthen the textural and sensorial properties of the cupcake. The dependent variables were baking loss, specific volume, textural characteristics (hardness, springiness, cohesiveness, gumminess, chewiness, resilience), and number of pores. The parameters' lower and upper limits were determined by prior baking trials, as shown in Table 2.

**TABLE 2:** Levels of independent variables for the cake production.

Independent variables		Levels		
Stevia, (%)	A	0	0.6	1.2
Psyllium, (%)	B	0	2	4
Baking type	C	Air fryer/oven		

Using multiple regression, second‐order polynomial models have been matched to each of the dependent variables via the Design‐Expert v13 (Stat‐Ease) program. The overall experimental design included 26 trials, five of which were recurrence of the central point. Experimental analyses were fitted with the generalized model employed in RSM, as illustrated in Equation (3).

(3) $$\begin{array}{ccc} Y & = & \beta_{0}+\beta_{1}X_{1}+\beta_{2}X_{2}+\beta_{3}X_{3}+\beta_{11}X_{1}^{2}+\beta_{22}X_{2}^{2}+\beta_{33}X_{3}^{2}\\ & & +\;\beta_{12}X_{1}X_{2}+\beta_{13}X_{1}X_{3}+\beta_{23}X_{2}X_{3}\; \end{array}$$

In Equation (3), *Y* denotes variable responses, *β* denotes regression coefficients, and *X* denotes coded factors. RSM graphs' 3D plots are graphical representations of regression equations, displaying the correlation between independent and dependent variables. The analysis of variance (ANOVA), a more trustworthy method of assessing the quality of the model fitted, was used by Design‐Expert to optimize the process. Using Design‐Expert, an ANOVA was utilized to determine the individual linear, quadratic, and interaction regression coefficients. The coefficient of determination (*R* ^2^) was used to evaluate the polynomial equation's conformity to the responses. All polynomial equation terms were statistically examined using the *F* value at *p* < 0.05. The optimized conditions were validated for the maximum specific volume, springiness, pore numbers, and minimum hardness, cohesiveness, gumminess, chewiness, and resilience using the values acquired by RSM. Table 3 presented the independent variables (stevia, psyllium, baking type) with the experimental values of the dependent variables (baking loss, specific volume, hardness, springiness, cohesiveness, gumminess, chewiness, resilience, number of pores) as provided by RSM‐CCFD. Tables 4 and **5** illustrated the comprehensive results of the ANOVA models.

**TABLE 3:** Response Surface Methodology‐Central Composite Face‐Centered Design (RSM‐CCFD) employed for reformulation of gluten‐free sugar‐reduced cupcake.

	Independent variables			Dependent variables								
Run	Stevia	Psyllium	Baking type	Baking loss(%)	Specific volume(mL/g)	Hardness (gf)	Springiness	Cohesiveness	Gumminess	Chewiness	Resilience	Number of pore
1	1.2	2	Airfryer	6.45	28.12	2787.12	0.921	0.611	1397.12	1620.45	0.307	25
2	0.6	2	Airfryer	7.74	29.2	2477.45	0.933	0.64	1593.46	1477.74	0.327	28
3	0.6	2	Airfryer	7.73	29.45	2476.34	0.933	0.642	1590.52	1480.41	0.326	22
4	0.6	2	Airfryer	7.74	29.52	2478.06	0.932	0.641	1592.74	1485.63	0.328	26
5	0	4	Airfryer	7.41	33.15	2428.25	0.938	0.639	1549.35	1449.92	0.34	32
6	1.2	4	Airfryer	5.43	31.23	3025.79	0.939	0.654	1978.72	1857.23	0.336	45
7	1.2	0	Airfryer	5.07	24.75	4310.75	0.918	0.601	2588.16	2376.21	0.285	19
8	0.6	0	Airfryer	6.75	26.18	3420.16	0.925	0.634	2164.15	2002.96	0.3165	23
9	0	0	Airfryer	9.77	22.46	3011.45	0.926	0.633	1904.87	1764.74	0.3245	25
10	0.6	4	Airfryer	7.65	35.15	2105.45	0.935	0.6285	1323.79	1243.12	0.3275	29
11	0.6	2	Airfryer	7.73	29.45	2477.74	0.933	0.642	1593.42	1480.63	0.324	28
12	0.6	2	Airfryer	7.74	29.39	2478.65	0.931	0.642	1590.1	1487.89	0.328	27
13	0	2	Airfryer	7.93	32.23	2152.2	0.94	0.656	1453.61	1366.33	0.349	36
14	0	4	Oven	8.89	29.35	1912.45	0.942	0.691	1440.20	1386.28	0.375	51
15	1.2	2	Oven	5.3	27.17	2378.47	0.94	0.66	1568.45	1474.41	0.347	59
16	0.6	2	Oven	7.5	36.12	1648.45	0.938	0.661	1087.40	1012.36	0.35	41
17	0.6	2	Oven	7.51	36.23	1646.74	0.94	0.66	1085.48	1015.45	0.352	42
18	0.6	2	Oven	7.49	36.45	1645.32	0.939	0.662	1082.74	1019.1	0.351	39
19	0	2	Oven	7.73	30.56	1664.52	0.935	0.654	1309.63	1247.45	0.331	46
20	0.6	2	Oven	7.51	36.32	1644.9	0.938	0.662	1085.23	1029.45	0.361	40
21	1.2	4	Oven	5.05	34.68	4068.79	0.938	0.657	2329.95	2183.28	0.332	23
22	0.6	0	Oven	9.07	33.2	2567.17	0.925	0.637	1635.41	1512.96	0.3325	15
23	0	0	Oven	9.55	20.85	2040.32	0.927	0.648	1319.45	1222.83	0.348	17
24	1.2	0	Oven	5.21	33.75	3087.62	0.926	0.616	1900.13	1760.41	0.308	20
25	0.6	4	Oven	6.87	33.15	2149.78	0.931	0.664	1352.46	1260.42	0.351	47
26	0.6	2	Oven	7.51	36.48	1648.97	0.938	0.659	1087.41	1018.45	0.351	42

**TABLE 4:** Model summary and analysis of variance (ANOVA) results for the outcomes of baking loss, specific volume, hardness, springiness and cohesiveness.

	Sum of squares					Mean squares					*F*‐Value					*p*‐Value				
Source	Baking loss	Specific volume	Hardness	Springiness	Cohesiveness	Baking loss	Specific volume	Hardness	Springiness	Cohesiveness	Baking loss	Specific volume	Hardness	Springiness	Cohesiveness	Baking loss	Specific volumev	Hardness	Springiness	Cohesiveness
Model	37.38	387.08	1.209 × 10^7^	0.0009	0.0075	3.40	35.19	1.100 × 10^6^	0.0001	0.0007	10.77	5.77	21.51	6.80	6.27	<0.0001	0.0015	<0.0001	0.0006	0.0010
A‐Stevia	29.36	10.27	3.466 × 10^6^	0.0001	0.0012	29.36	10.27	3.466 × 10^6^	0.0001	0.0012	93.03	1.68	67.80	4.66	11.42	<0.0001	0.2154	<0.0001	0.0486	0.0045
B‐Psyllium	1.41	105.14	6.288 × 10^5^	0.0005	0.0023	1.41	105.14	6.288 × 10^5^	0.0005	0.0023	4.48	17.24	12.30	39.86	20.76	0.0526	0.0010	0.0035	<0.0001	0.0004
C‐Baking type	0.1430	100.49	1.748 × 10^6^	0.0001	0.0011	0.1430	100.49	1.748 × 10^6^	0.0001	0.0011	0.4532	16.48	34.18	7.59	10.35	0.5118	0.0012	<0.0001	0.0155	0.0062
AB	1.30	17.35	20,734.62	4.500 × 10^−6^	0.0003	1.30	17.35	20,734.62	4.500 × 10^6^	0.0003	4.11	2.84	0.4056	0.3726	2.33	0.0622	0.1138	0.5345	0.5513	0.1491
AC	0.5002	28.77	1.600 × 10^5^	0.0001	3.333 × 10^−7^	0.5002	28.77	1.600 × 10^5^	0.0001	3.333 × 10^−7^	1.58	4.72	3.13	4.66	0.0031	0.2286	0.0475	0.0986	0.0486	0.9566
BC	0.3072	23.41	1.091 × 10^6^	8.333 × 10^−6^	0.0003	0.3072	23.41	1.091 × 10^6^	8.333 × 10^−6^	0.0003	0.9734	3.84	21.35	0.6901	2.54	0.3406	0.0703	0.0004	0.4201	0.1335
A^2^	2.38	58.17	4.972 × 10^5^	1.681 × 10^−6^	0.0000	2.38	58.17	4.972 × 10^5^	1.681 × 10^−6^	0.0000	7.54	9.54	9.73	0.1392	0.2695	0.0158	0.0080	0.0075	0.7146	0.6118
B^2^	0.0319	3.95	2.090 × 10^6^	0.0001	0.0002	0.0319	3.95	2.090 × 10^6^	0.0001	0.0002	0.1012	0.6470	40.88	9.05	2.27	0.7551	0.4346	<0.0001	0.0094	0.1544
ABC	0.6161	1.41	4.099 × 10^5^	0.0000	0.0003	0.6161	1.41	4.099 × 10^5^	0.0000	0.0003	1.95	0.2314	8.02	1.49	2.76	0.1841	0.6379	0.0133	0.2423	0.1187
A^2^C	0.3152	29.45	50,852.86	0.0000	0.0000	0.3152	29.45	50,852.86	0.0000	0.0000	0.9988	4.83	0.9947	0.9143	0.1211	0.3346	0.0453	0.3355	0.3552	0.7330
B^2^C	1.29	0.8792	76,738.98	0.0001	1.870 × 10^6^	1.29	0.8792	76,738.98	0.0001	1.870 × 10‐^6^	4.09	0.1442	1.50	4.36	0.0172	0.0626	0.7099	0.2407	0.0556	0.8975
Residual	4.42	85.38	7.157 × 10^5^	0.0002	0.0015	0.3156	6.10	51,122.46	0.0000	0.0001										
Baking loss(%)					Specific volume				Hardness				Springiness				Cohesiveness			
*R* ^2^: 0.89. Adj *R* ^2^: 0.81. CV: 7.67 Standard deviation: 0.56. Mean: 7.32					*R* ^2^: 0.81. Adj *R* ^2^: 0.67. CV: 7.98 Standard deviation: 2.47 Mean: 30.95				*R* ^2^: 0.94. Adj *R* ^2^: 0.90. CV: 9.22 Standard deviation: 226.10 Mean: 2451.27				*R* ^2^: 0.84. Adj *R* ^2^: 0.71. CV: 0.37 Standard deviation: 0.0035 Mean: 0.9331				*R* ^2^: 0.83. Adj *R* ^2^: 0.70. CV: 1.61 Standard deviation: 0.0104 Mean: 0.6459			

Abbreviation: CV, coefficient of variation.

**TABLE 5:** Model summary and analysis of variance (ANOVA) results for the outcomes of gumminess, chewiness, resilience and number of pores.

	Sum of squares				Mean square				*F*‐value				*p*‐value			
Source	Gumminess	Chewiness	Resilience	Number of pores	Gumminess	Chewiness	Resilience	Number of pores	Gumminess	Chewiness	Resilience	Number of pores	Gumminess	Chewiness	Resilience	Number of pores
Model	3.352 × 10^6^	2.935 × 10^6^	0.0066	2800.27	3.047 × 10^5^	2.669 × 10^5^	0.0022	254.57	8.88	14.02	19.67	6.25	0.0002	<0.0001	<0.0001	0.0010
A‐Stevia	6.465 × 10^5^	6.695 × 10^5^	0.0019	21.33	6.465 × 10^5^	6.695 × 10^5^	0.0019	21.33	18.83	35.16	17.43	0.5239	0.0007	<0.0001	0.0004	0.4811
B‐Psyllium	1.970 × 10^5^	1.323 × 10^5^	0.0018	972.00	1.970 × 10^5^	1.323 × 10^5^	0.0018	972.00	5.74	6.95	16.19	23.87	0.0311	0.0196	0.0006	0.0002
C‐Baking type	5.452 × 10^5^	5.105 × 10^5^	0.0028	804.44	5.452 × 10^5^	5.105 × 10^5^	0.0028	804.44	15.88	26.81	25.40	19.76	0.0014	0.0001	<0.0001	0.0006
AB	380.19	381.71	–	18.00	380.19	381.71	–	18.00	0.0111	0.0200	–	0.4420	0.9177	0.8894	–	0.5169
AC	37,753.06	6942.75	–	5.33	37,753.06	6942.75	–	5.33	1.10	0.3646	–	0.1310	0.3121	0.5556	–	0.7228
BC	3.581 × 10^5^	3.096 × 10^5^	–	75.00	3.581 × 10^5^	3.096 × 10^5^	–	75.00	10.43	16.26	–	1.84	0.0061	0.0012	–	0.1962
A^2^	1.645 × 10^5^	2.428 × 10^5^	–	105.94	1.645 × 10^5^	2.428 × 10^5^	–	105.94	4.79	12.75	–	2.60	0.0461	0.0031	–	0.1291
B^2^	7.131 × 10^5^	4.562 × 10^5^	–	410.51	7.131 × 10^5^	4.562 × 10^5^	–	410.51	20.77	23.96	–	10.08	0.0004	0.0002	–	0.0067
ABC	39,619.72	26,863.30	–	312.50	39,619.72	26,863.30	–	312.50	1.15	1.41	–	7.67	0.3009	0.2547	–	0.0150
A^2^C	97,148.07	43,397.47	–	0.0591	97,148.07	43,397.47	–	0.0591	2.83	2.28	–	0.0015	0.1147	0.1534	–	0.9701
B^2^C	3.20	7436.54	–	389.44	3.20	7436.54	–	389.44	0.0001	0.3906	–	9.56	0.9924	0.5421	–	0.0079
Residual	4.806 × 10^5^	2.666 × 10^5^	0.0024	570.07	34,331.08	19,040.26	0.0001	40.72			–	–	–	–	–	–
Gumminess					Chewiness				Resilience				Number of pores			
*R* ^2^: 0.87. Adj *R* ^2^: 0.77. CV: 11.86 Standard deviation: 185.29 Mean: 1561.69					***R* ^2^**: 0.91. **Adj *R* ^2^**: 0.85. **CV**: 9.38 **Standard deviation**: 137.99 **Mean**: 1470.62				***R* ^2^**: 0.72**. Adj *R* ^2^**: 0.70**. CV**: 3.15 **Standard deviation**: 0.0105 **Mean**: 0.3349				***R* ^2^**: 0.83**. Adj *R* ^2^**: 0.70**. CV**: 19.59 **Standard deviation**: 6.38 **Mean**: 32.58			

Abbreviation: CV, coefficient of variation.

## 3 RESULTS AND DISCUSSION

### 3.1 Baking loss

Sucrose is beneficial for sweet baked goods such as cakes as it helps to incorporate air bubbles into the dough during mixing. Sugar's water‐holding capacity results in dough softening, viscosity reduction, and slower gelatanization of starch in limited amounts (Schiatti‐Sisó et al., 2023). Baking leads to increased vapor pressure as the liquid expands when heated. The loss of gas during baking is known as “baking loss” (Singh et al., 2019). In this present study, the weight loss of the cupcakes during baking ranged from 5.05% to 9.77% (Table 3). The highest weight loss, 9.77%, was recorded when no stevia or psyllium was added to the air fryer baking method. Conversely, the lowest weight loss (5.05%) was observed when psyllium and stevia were added in the largest amounts, 1.2% and 4%, respectively, when baking in an oven. Similar to the present study, stevia acted as a humectant and prevented water loss during baking in muffin samples (Gao et al., 2019). The humectant properties of various sugar substitutes, including stevia, xylitol, inulin, and corn syrup, were evaluated by Samakradhamrongthai and Jannu (2021). Xylitol was found to be the most effective due to its higher water activity and providing a softer texture in candy items. Another study emphasized the need for careful integration of stevia into intermediate‐moisture bakery products, as its humectant properties could significantly contribute to a higher risk of fungal spoilage (Rodríguez et al., 2016). Additionally, the humectant mechanism of 5% stevia in a skin moisturizer was found to be highly effective in enhancing skin hydration (Kuntal Das et al., 2012). Consistent with the literature, present research demonstrated the humectant properties of stevia, which had a notable reducing effect on baking loss in both baking methods. On the other hand, the previous studies stated that complete replacement of sucrose with stevia in bakery products is not acceptable due to the poor bulking characteristics. In order to determine the significant effects of independent variables on the responses and the responses that were significantly affected by the varying processing conditions, ANOVA was conducted. ANOVA findings revealed that the reduced cubic model is statistically significant, with Fisher's *F*‐test value of 10.77 and *p*‐value of <0.0001 (Table 4). The *R* ^2^, adjusted *R* ^2^, and coefficient of variation (CV) values in this investigation were 0.89, 0.81, and 7.67, respectively. According to statistical examination of the baking loss, only the linear and quadratic terms of *A* and *A* ^2^ were considerably important at 95% confidence level, whereas the others had no significant influence statistically. The linear coefficient term (−1.56) of *A* had the most profound and negative effect on baking loss, as evidenced by the regression Equation (4). This indicated that increasing the amount of stevia reduced baking loss.

Equation (4) describes the link between baking loss (*Y*_1_) and the coded variables stevia (*A*), psyllium (*B*), and baking type (*C*).

(4) $$\begin{array}{ccc} Y_{1} & = & 7.59-1.56\;A-0.34\times B-0.11\times C+0.40\times AB\\ & & -\;0.20\times AC-0.16\times BC-0.65\times A^{2}+0.07\times B^{2}\\ & & -\;0.27\times ABC-0.23\times A^{2}\;C+0.48\times B^{2}C \end{array}$$

**FIGURE 1:**
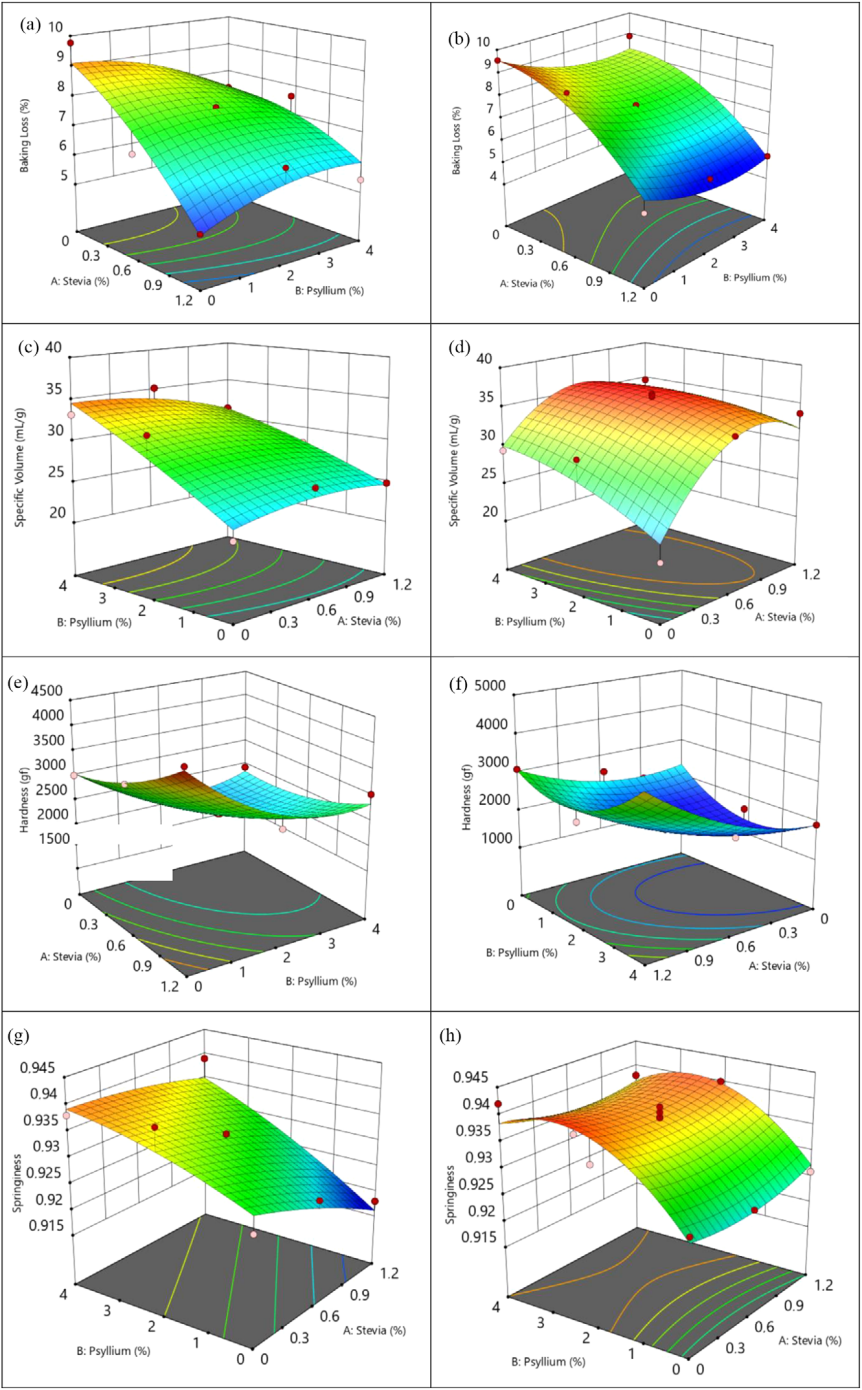
Baking loss (a, b), specific volume (c, d), hardness (e, f), and springiness (g, h) of response surface 3D plots for the interaction factors of stevia, psyllium, and the type of baking (left column: air fryer, right column: oven, red dots: experimental runs.).

Interaction effect of stevia, psyllium, and baking type on baking loss of cupcakes is clearly seen in Figure 1. There was a slight increase in air fryer baking and a small reduction in oven baking up to a certain point when psyllium was added. However, the baking loss was restricted by adding stevia. Different baking methods and parameters give rise to distinct cake products with different physical qualities. According to Sani et al. (2014), as an outcome of the air flow, the temperature is distributed more uniformly within the oven. When comparing the cakes’ weight loss with and without airflow, thermal gradient of the cakes was higher in airflow baking, thus enhancing the migration of moisture and resulting in more baking loss (Shahapuzi et al., 2015b).

According to Figure 1a, baking loss was greater overall, particularly at lower stevia and psyllium concentrations. However, it steadily decreased as the stevia and psyllium concentrations increased. Figure 1b demonstrated a more significant reduction in baking loss as the amount of stevia increased. In addition, the lowest baking loss was observed at 2% psyllium in oven baking, as illustrated in Figure 1b. It is because of water‐binding characteristics of psyllium as reported by previous researchers. It has been observed that psyllium addition results in more humid and softer bread (Belorio & Gómez, 2021). Man et al. (2017) reported that adding 15% psyllium enhances the moisture content of breads by approximately 25%, while Krystyjan et al. (2018) recommended adding 3%–20% psyllium into cookies. According to Saeidy et al. (2023), the addition of gums contributed to preserving the moisture content and density of gluten‐free muffins. Specifically, moisture retention was greatly increased by locust bean gum and pectin derived from vegetable and fruit fibers. It is evident in both figures (Figure 1a,b) that higher concentrations of stevia and a moderate percentage of psyllium are effective in reducing baking loss. However, the impact is more significant in an air fryer. Table 3 demonstrated that the maximum and minimum baking loss for the oven baking method is ranged from 9.55% to 5.05%, respectively. Meanwhile, for air fryer baking, it ranged from 9.77% to 5.07%. The reduction in baking loss that results from increasing stevia and psyllium concentrations is more pronounced in the air fryer baking method. In accordance with the current investigation, Ngan et al. (2023) reported that air fryer baking led to a greater weight loss (22.2%) than oven baking (20.5%), which in turn resulted in a greater moisture loss. Similar to this study, replacing sugar with *Stevianna* preserved the moisture content of the muffin samples while acting as a humectant during baking (Gao et al., 2019). This could be attributed to the rapid air circulation of air fryer baking, whereas oven baking may be more effective in holding moisture, particularly when psyllium and stevia are used as they exhibit water‐binding properties.

Moisture contents of optimized and control cake batter were determined to be 45.08 ± 0.19% and 31.95 ± 0.77%, respectively. These findings validated the moisture retention properties of psyllium and stevia in bakery items. Additionally, the optimized cake exhibited a moisture content of 30.93 ± 0.61% when baked in the oven and 26.92 ± 0.05% when baked in the air fryer. In contrast, the control cake had a moisture content of 21.75 ± 0.87%. Furthermore, the final moisture levels of the cakes were significantly influenced by the baking technique, with the air fryer method resulting in a greater reduction in moisture than oven baking.

### 3.2 Specific volume

The specific volume of the gluten‐free sugar‐reduced cupcakes in the current study varied from 20.85 to 36.48 mL/g (Table 3). The highest specific volume (36.48 mL/g) was recorded at 0.6% stevia, 2% psyllium for oven baking, and 0.6% stevia and 4% psyllium for air frying (35.15 mL/g) baking processes. Also, the lowest values were 20.85 and 22.46 mL/g for air fryer and oven, respectively, under no addition of stevia and psyllium. Lack of gluten is a key cause of quality, structure, and shelf life problems with bakery goods (Khoury et al., 2018). Researchers have stated that psyllium may work as a gluten replacer, mimicking the properties of gluten to enhance the volume, texture, and overall acceptance of gluten‐free products (Fratelli et al., 2018). The ANOVA results (Table 4) showed that the reduced cubic model is well fitted with a *p*‐value of 0.0015 for describing the parameters on specific volume of gluten‐free cupcakes. Also, the model's *F*‐test value is 5.77. The linear effects of *B* and *C*, the quadratic effect of *A* ^2^, and the interaction effects of *AC* and *A* ^2^ *C* are all significant (*p* > 0.05) in the suggested model. Given that the low CV values of the research are signs of its trustworthiness and accuracy. In the current study, the CV value was determined to be 7.98%. Statistically, psyllium was the most significant term among the others (*p*‐value = 0.001), while *AC* was the least significant (*p*‐value = 0.0475). It was consistent with the sum of squares results that the importance of variables appeared as *B* > *C* > *A* ^2^. The *R* ^2^ and adjusted *R* ^2^ values of 0.81 and 0.67, respectively, confirmed the model's adequacy in this study.

Equation (5) was determined in order to assess the impact of psyllium (*B*), baking type (*C*), and stevia (*A*) on the specific volume (*Y*_2_).

(5) $$\begin{array}{ccc} Y_{2} & = & 32.83+0.92\times A+2.96\times B+2.94\times C-1.47\times AB\\ & & +\;1.55\times AC-1.40\times BC-3.24\times A^{2}-0.84\times B^{2}\\ & & -\;0.42\times ABC-2.30\times A^{2}C-0.39\times B^{2}C \end{array}$$

Figure 1c,d illustrated that the specific volume of gluten‐free cakes was significantly impacted by the type of baking, with oven baking producing a higher volume than air frying baking in general. In order to assess the influence of two baking methods on the specific volume of the cakes, runs 9 and 23 provided information due to the nonexistence of stevia and psyllium. In run 23, the specific volume of oven‐baked cake (20.85 mL/g) was less than that of air‐fried cake in run 9 (22.46 mL/g).

As stated by Mior Zakuan Azmi et al. (2019), the use of an air fryer for baking led to a slightly higher cake height compared to using an oven. This can be attributed to the enhanced air circulation and increased convective heat transfer in the air fryer. Baking temperature and time significantly influenced cake height in both baking methods (*p* < 0.05), according to Mior Zakuan Azmi et al. (2019). Azmi et al. (2020) reported that the rapid air circulation and enhanced convective heat transfer in an air fryer promote greater volume expansion. Thus, cakes baked in an air fryer exhibited faster expansion compared to those baked in an oven. Furthermore, the high heating rate of the air fryer accelerated structural changes, leading to a firmer cake crumb, a burnt texture together with volume expansion (Mior Zakuan Azmi et al., 2019). The findings of the present study completely aligned with the literature that air‐fried cakes had a higher specific volume due to the air fryer's rapid heating process.

Figure 1c showed that in air frying baking, the specific volume of the cupcakes gradually increased when the amount of psyllium was increased. Similarly, adding 2.86% psyllium to flour weight in gluten‐free bread boosted specific volume by over 50% compared to the control. However, further increases in psyllium resulted in a decrease in specific volume (Fratelli et al., 2021). It is claimed that the addition of ingredients such as psyllium and hydroxymethylcellulose (HPMC) enhanced the viscosity of the gluten‐free dough and strengthened the walls of enlarging cells during baking. This causes the dough to trap more gas and become voluminous. Additionally, in this present study, the effect of stevia was minimal in air fryer baking, as the plots showed approximately plateau. It was corresponding with the work of Sulaiman et al. (2022) that as the replacing level of sugar increased with stevia leaves powder or stevia aqueous extract, height and specific volume of the cake gradually decreased. In addition, the specific volume of traditional Malaysian cake decreased when sugar was replaced with stevia, resulting in the lowest ratio of 50% stevia and 50% isomalt (Hamzah et al., 2013). As observed in Figure 1d, the 3D plots reached high levels during oven baking at moderate stevia concentrations and greater psyllium percentages. Moreover, stevia had a more powerful impact on specific volume in the oven baking process. Nevertheless, as the stevia concentrations increased, the volume appeared to remain stable or even slightly decrease.

### 3.3 Hardness

The peak force that was measured during the initial compression was described as hardness value (Gökçe et al., 2023). Previous research states that hardness generally showed the same trend with density of the product and opposite trend with the volume of the product (Salehi & Kashaninejad, 2018). Less voluminous cakes were smaller, denser, and harder in structure (Aslan Türker et al., 2023). The hardness of the gluten‐free cupcakes in this investigation varied from 1644.90 to 4310.75 gf (Table 3). This finding aligned with the hardness of gluten‐free cake produced with taro flour, ranging from 1714.97 to 4514.78 gf (Ekafitri et al., 2019). The lowest hardness was obtained via oven baking with 0.6% stevia and 2% psyllium, whereas the highest hardness was obtained with air fryer baking with 1.2% stevia and no psyllium. Air fryer‐baked items tend to have a higher overall hardness than oven‐baked products. This is a consequence of the air fryer's rapid air circulation and elevated temperature, which facilitates heat transfer and leads to moisture loss that increases the product's hardness (Murzaini et al., 2020). A predictive model of significance with an *F*‐test value of 21.51 and a *p*‐value <0.0001 was shown by the ANOVA outcomes in Table 4 for hardness. In addition to the quadratic terms of *A* ^2^ and *B* ^2^, and the interaction terms of *BC* and *ABC*, stevia, psyllium, and baking type all have significant effect on hardness. Each variable's significance was evaluated using the *p*‐value. As the *p*‐value dropped, the related coefficient became more important. The most profound terms were stevia, baking type, and quadratic terms of psyllium (*B* ^2^) with the lowest *p*‐values (<0.0001). High *R* ^2^, adjusted *R* ^2^, and low CV values of 0.94%, 0.90%, and 9.22% reinforced the model's adequacy, appropriateness, and reproducibility. According to regression Equation (4), all coefficient components, with the exception of the linear terms for psyllium and baking type, have a positive impact on hardness. Negative values state that increase causes reduction in hardness. In the regression equation, term *B* ^2^ had the greatest and positive value, showing the strongest influence on the response variable. Equation (6) illustrates the correlation between hardness (*Y*_3_) and the independent variables stevia (*A*), psyllium (*B*), and baking type (*C*) through coded variables.

(6) $$\begin{array}{ccc} Y_{3} & = & 2028.92+537.44\times A-228.91\times B-388.14\times C\\ & & +\;50.91\times AB+115.48\times AC+301.56\times BC+300\\ & & \times \;A^{2}+615.07\times B^{2}+226.35\times ABC+95.94\times A^{2}C\\ & & +\;117.86\times B^{2}C \end{array}$$

Figure 1e,f illustrates that psyllium had a favorable effect on lowering hardness in air fryer baking process, particularly at moderate to high incorporation levels. It was explained by Biliaderis et al. (1995) that hardness reduction in bread was deeply related with the increasing amount of arabinoxylan, which is the main component of psyllium. Consistent with this finding, the minimal crumb hardness of gluten‐free bread with psyllium addition was linked to the water retention capacity of psyllium fibers all during the baking process (Filipčev et al., 2021). Nevertheless, the 3D plot line's hardness was distinctly concave at oven baking in Figure 1f, with the lowest hardness located in the middle and the highest hardness at the edges. It was reported by Beikzadeh et al. (2016) that the hardness of sponge cake increased as the addition level of psyllium husk increased from 10% to 15%. The lowest hardness recorded was 7.5%, and the maximum hardness was 15%. Likewise, the addition of psyllium resulted in an increase in the hydration properties of the starch solution and a decrease in its hardness. Furthermore, in both figures (Figure 1e,f), increasing stevia from 0.6% to 1.2% raises cupcake hardness, particularly at high concentrations, but the effect is greater in air fryer baking. As evidenced by the same trend, the muffin sample's hardness increased by 1.5 times when the steviol glycosides substituted sucrose from the control to 100% (Karp et al., 2016).

### 3.4 Springiness

Springiness is a way to measure elasticity, which is an object's ability to recover its original height after being compressed. After the force is withdrawn, the distance at which the deformed cake returns to its non‐deformed state is used to measure springiness (Gökçe et al., 2023). Chandra and Shamasundarn (2015) stated that high springiness necessitates more chewing effort. In the current investigation, the springiness of the gluten‐free cake varied from 0.918 to 0.942 (Table 3). This outcome aligns with earlier research (Gökçe et al., 2023; Salehi & Kashaninejad, 2018). In the current study, air fryer baking with 1.2% stevia and no psyllium addition resulted in the lowest springiness, while oven baking with 4% psyllium and no stevia addition yielded the highest springiness. The ANOVA results in Table 4 exhibit that the model proposed for springiness was primarily influenced by all linear elements of stevia, psyllium, and baking type, along with the interaction term of *AC* and the quadratic term of *B* ^2^. Psyllium was the most significant term, with the lowest *p*‐value (<0.0001) and highest magnitude (0.0063) in the mathematical equation. According to the ANOVA data, a reduced cubic model was offered with a *p*‐value of 0.0006 and an *F*‐test value of 6.80. The current study achieved an *R* ^2^ of 0.84, an adjusted *R* ^2^ of 0.71, and a CV value of 0.37%. In Equation (7), the relationship between springiness (*Y*_4_) and the independent variables of stevia (*A*), psyllium (*B*), and baking type (*C*) of coded variables was specified.

(7) $$\begin{array}{ccc} Y_{4} & = & 0.93-0.0021\times A+0.0063\times B+0.0028\times C\\ & & +\;0.00075\times AB+0.0022\times AC-0.00083\times BC\\ & & +\;0.0006\times A^{2}-0.0044\times B^{2}-0.0015\times ABC\\ & & +\;0.0014\times A^{2}C-0.003\times B^{2}C \end{array}$$

The springiness of both baking methods was enhanced by the use of psyllium, as illustrated in Figure 1g,h. However, in oven baking, the springiness remained higher at a higher concentration of psyllium and decreased more sharply as the concentration of psyllium decreased in contrast to air fryer baking. As the psyllium concentration increased, springiness increased positively and steadily, as seen in Figure 1g. In a study, oat fiber has been added to rice flour and corn flour‐based gluten‐free cakes. The cake's springiness increased profoundly in both circumstances (Karp et al., 2019). In contrast to this observation, increasing quince powder replacement from 0% to 15% reduced sponge cake's springiness (Salehi & Kashaninejad, 2018). In contrast, the air fryer baking of the cupcakes resulted in a minor increase in springiness as the quantity of stevia dropped (Figure 1h). In accordance with the research conducted by Majzoobi et al. (2018), the springiness of the cakes increased from 0.96 to 1 as the replacement of sucrose increased from 0% to 100% with stevia and inulin. As seen in Figure 1h, stevia did not affect springiness property when baked in the oven.

### 3.5 Cohesiveness

Cohesiveness indicates the internal perseverance of food structure. The capacity of a material to remain attached to itself is called cohesiveness. It referred to how effectively the product resists a second deformation in comparison to its resilience to the initial deformation (Salehi & Kashaninejad, 2018). In the current study, cohesiveness of the samples changed from 0.601 to 0.656 in the air fryer baking method and 0.616–0.691 in the oven baking method (Table 3). It was in line with the cohesiveness of protein‐enriched gluten‐free layer cakes, which were in the range of 0.54 and 0.64. The minimum cohesiveness was obtained in air fryer baking method at 1.2% stevia and no psyllium, while the highest cohesiveness was obtained with 0% stevia and 4% psyllium by oven baking. For the cohesiveness of gluten‐free cupcakes, ANOVA proposed a reduced cubic model (Table 4) with *F*‐value of 6.27 and *p*‐value of 0.001. The *R* ^2^, adjusted *R* ^2^, and CV values were 0.83%, 0.69%, and 1.61%, respectively. In the present study, only the linear terms of stevia, psyllium, and baking type were significant (*p* < 0.05). As seen in the regression Equation (8), all coefficient terms have a positive effect on cohesiveness except for the linear terms of stevia, the quadratic terms of *A* ^2^, and the intreraction terms of *ABC* and *B* ^2^ *C*. In the mathametical equation, negative values cause reduction while positive values raise response. Psyllium had the highest and most effective parameter with the lowest *p*‐value (0.0004) and equivalently highest coefficient (0.014), as can be seen from Equation (8).

The relationship between cohesiveness (*Y*_5_) and the independent variables stevia (*X*_1_), psylliume (*X*_2_), and baking type (*X*_3_) of coded variables were demostrated in Equation (8).

(8) $$\begin{array}{ccc} Y_{5} & = & 0.65-0.01\times A+0.014\times B+0.0098\times C+0.0056\\ & & \times \;AB+0.0002\times AC+0.0048\times BC-0.0023\times A^{2}\\ & & -\;0.006\times B^{2}-0.006\times ABC+0.0015\times A^{2}C\\ & & -\;0.0006\times B^{2}C \end{array}$$

**FIGURE 2:**
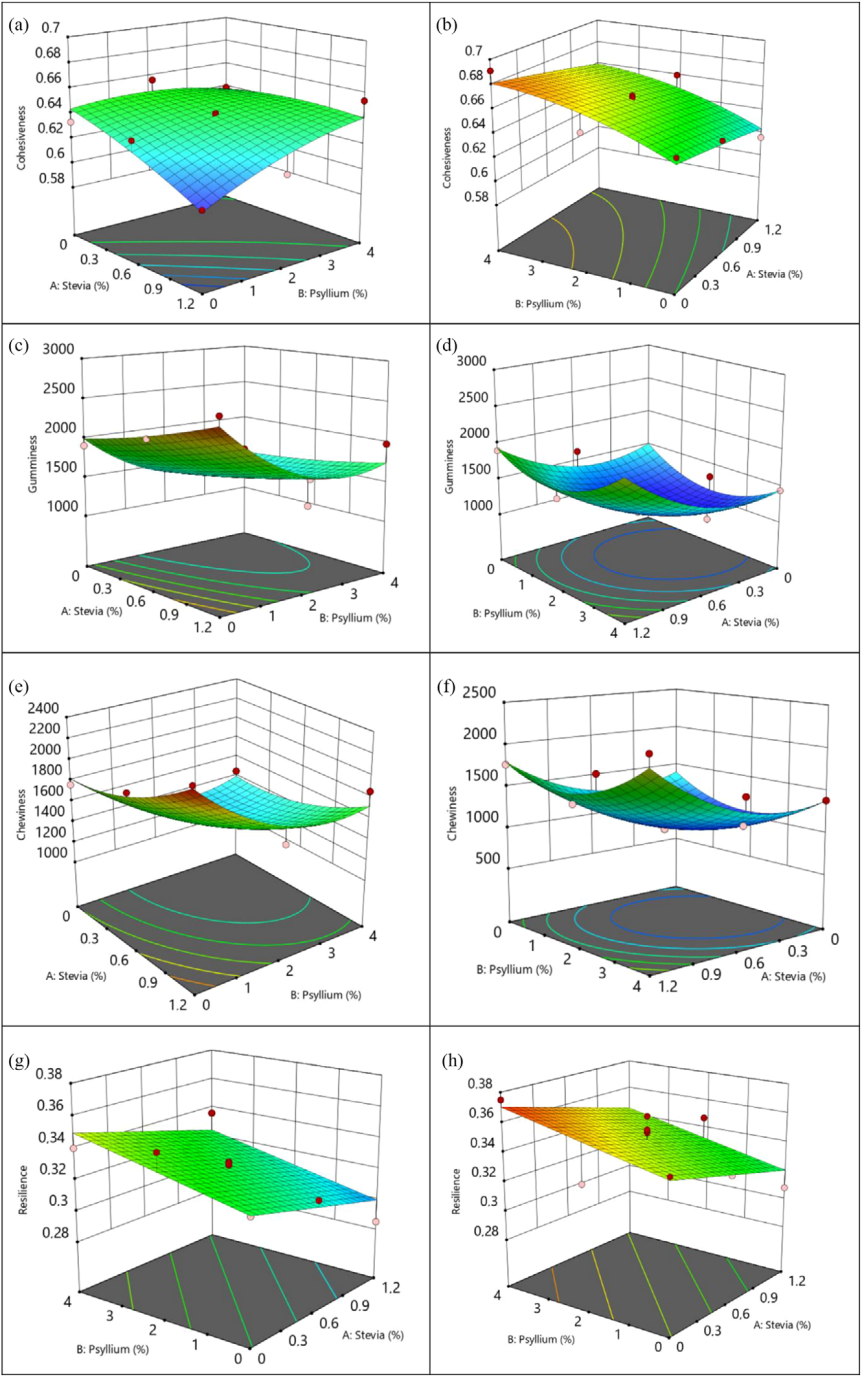
Cohesiveness (a, b), gumminess (c, d), chewiness (e, f), and resilience (g, h) of response surface 3D plots for the interaction factors of stevia, psyllium, and the type of baking (left column: airfryer, right column: oven, red dots: experimental runs.).

Figure 2a,b showed how psyllium, stevia, and baking methods influenced the cohesiveness of gluten‐free cupcakes. As can be observed, the oven produced gluten‐free cupcakes with a higher cohesiveness than the air fryer baking method. According to Al‐Muhtaseb et al. (2013), cohesiveness increased with increasing moisture content in the cakes. It can be related to baking methods, temperature, and time. The results of the current study supported these findings, as rapid air circulation in the air fryer accelerated moisture loss and resulted in less cohesive cupcakes. The addition of psyllium increases cohesiveness in both ways, despite the impact being greater in oven baking (Figure 2b). Parallel to this, adding psyllium at a rate of 1%–10% increased the cohesiveness of the bread sample from 0.78 to 0.84 as reported by M. Franco and Gómez (2022). As shown in Figure 2a, increasing stevia decreased cupcake cohesiveness in air fryer baking but had no effect in oven baking. This is consistent with the findings of Manisha et al. (2012), who found that replacing sugar with stevia and liquid sorbitol reduced cake cohesiveness. However, the contrary results showed that stevia inclusion resulted in a slight increase in cupcake cohesiveness (Quitral et al., 2019).

### 3.6 Gumminess

Gumminess shows the amount of energy necessary to disintegrate the cake to a state suitable for swallowing. Hardness and cohesiveness are multiplied to get gumminess (Jahanbakhshi & Ansari, 2020). The increased gumminess is a result of a higher hardness value. According to Chandra and Shamasundar (2015), gumminess is a quality of semisolid foods that have a high cohesion and a low hardness. The gumminess of the samples in the current study ranged from 1082.74 to 2588.16, as shown in Table 3. The air fryer cake formulation with 1.2% stevia and 0% psyllium exhibited the highest gumminess, while the oven baking formulation with 0.6% stevia and 2% psyllium exhibited the lowest gumminess. The results in ANOVA Table 5 indicate that all linear components of *A*, *B*, and *C*, as well as the quadratic terms *A* ^2^ and *B* ^2^, were significantly important (*p* < 0.05). The only significant term was the interaction effect of *BC*. Based on regression analysis, the reduced cubic model (*F*‐value of 8.88 and *p*‐value of 0.0002) is a significant predictor of gumminess values of gluten‐free cupcakes. As the lower *p*‐values demonstrated the higher significance, the most effective terms were ordered as follows: *B* ^2^ > *A* > *C*. The importance of the model equation and its level of fitness are assessed by examining the *R* ^2^ and adjusted *R* ^2^ values. In the current investigation, the values of *R* ^2^, adjusted *R* ^2^, and CV% were 0.87, 0.77, and 11.86, respectively. Because the *R* ^2^ values were rather high, the suggested model could be successful in estimating the gumminess of the cupcakes when the independent variables were combined. The mathematical model shown in Equation (9) indicates that only the negative coefficient terms of *B* and *C* exhibit a decreasing effect, whereas the remaining terms possess a positive and enhancing effect on the corresponding response. The *B* ^2^ (359.30) coefficient displayed the most significant influence on the response according to the equation. The correlation between gumminess (*Y*_6_) and the independent variables of stevia (*A*), psyllium (*B*), and baking style (*C*) is illustrated in Equation (9).

(9) $$\begin{array}{ccc} Y_{6} & = & 1316.22+232.12\times A-128.14\times B-216.79\times C\\ & & +\;6.89\times AB+56.09\times AC+172.74\times BC+172.55\\ & & \times \;A^{2}+359.3\times B^{2}+70.37\times ABC+132.61\times A^{2}C\\ & & +\;0.76\times B^{2}C \end{array}$$

Upon examination of figures (Figure 2c,d), it is evident that oven baking tends to produce less gumminess than air fryers. The gumminess values were lower when the psyllium concentration was 2% in both baking methods. However, Figure 2c demonstrated that lowering the psyllium concentration and increasing the stevia improved the gumminess of the cupcakes in air fryer baking. On the other hand, the gumminess was lowest in the medium percentages of psyllium, and it increased, especially with increasing addition of psyllium, indicating that psyllium addition had a curved impact in the oven baking method (Figure 2d). In a similar manner, the hardness, gumminess, and chewiness of the sponge cake elevated with the increasing incorporation of turmeric powder (Seo et al., 2010). Torres‐Perez et al. (2023) found that increasing the amount of psyllium increased the gumminess of gluten‐free bread baked in the oven. Nevertheless, Salehi and Kashaninejad (2018) found that the gumminess of the sponge cakes was increased by an increase in the level of quince powder replacement up to 10%, while a subsequent increase resulted in a reduction in gumminess. Figure 2c demonstrated that the impact of stevia in the air fryer baking method was more pronounced, achieved greater levels, and climbed with the amount added compared to the oven baking method. It might be explained by stevia's lower moisture retention capacity than sugar, which promptly increased the hardness, density, and gumminess of baking products (Quitral et al., 2019). Previous research of Hamzah et al. (2013) has confirmed that adding more stevia powder to Kuih Baulu, a traditional Malaysian cake, increased gumminess.

### 3.7 Chewiness

The force required to chew a firm substance until it is soft enough to swallow is referred to as chewiness. It is determined by multiplying gumminess and springiness (Lu et al., 2010). The investigation revealed that the chewiness of gluten‐free cupcakes varied between 2588 and 1323 when baked in air fryer, and between 1012 and 2183 when baked in oven as detailed in Table 3. According to ANOVA (Table 5), the regression coefficient of the model is statistically significant, with an *F*‐value of 14.02 and a *p*‐value of <0.0001. All linear and square variables of *A* ^2,^ *B* ^2^, and the combined effect of *BC* were statistically significant (*p* ≤ 0.05). The *R* ^2^, adjusted *R* ^2^, and CV values in the current study were 0.91, 0.85, and 9.38, respectively. *R* ^2^ values exceeding 0.75 signify the model's consistency and trustworthiness (Chaiya et al., 2015). The increase in significance of the corresponding coefficient in Equation (10) is reflected by the *p*‐value. The coefficient of the quadratic term *B* ^2^ (287.37) exhibited the largest value and a very low *p*‐value (0.0002). The negative coefficient values of *B* (−104.99) and *C* (−209.78) pointed to the independent variable C, which resulted in a greater reduction in the relevant response than B. Equation (10) clarifies the interaction between chewiness (*Y*_7_) and the independent variables stevia (*A*), psyllium (*B*), and baking type (*C*) in terms of coded variables.

(10) $$\begin{array}{ccc} Y_{7} & = & 1241.22+236.20\times A-104.99\times B-209.78\times C\\ & & +\;6.91\times AB+24.05\times AC+160.61\times BC+209.66\\ & & \times \;A^{2}+287.37\times B^{2}+57.95\times ABC+88.64\times A^{2}C\\ & & +\;36.69\times B^{2}C \end{array}$$

The combined effects of the independent variables of psyllium, stevia, and baking method (oven, air fryer) on chewiness were displayed in 3D plots of RSM graphs in Figure 2e,f. Baking methods were previously reported to be highly effective in enhancing the chewiness of cakes (Al‐Muhtaseb et al., 2013). In both baking methods, an increase in the level of stevia in the cake batter increased the chewiness of the final product (Figure 2e,f). In the air fryer baking method, chewiness increased as the percentage of stevia increased, and the concentration of psyllium decreased, as depicted in Figure 2e. Several research studies have consistently reported that an increase in stevia percentages enhanced chewiness (Hamzah et al., 2013; Kotebagilu et al., 2022). In a similar manner, the chewiness and hardness of the cassava cake exhibited an increase as the sugar concentration was elevated (Gan et al., 2007). It is believed that an excess of sugar may cause products to become more rigid because sugar controls the starch gelatinization after baking. Figure 2f demonstrated that with a rise in psyllium concentration, there is a slight reduction in chewiness. Psyllium plays a role in the reduction of chewiness to a certain extent; however, the rapid air mechanism of the air fryer limited its maximum potential. Considering the stevia's increasing effect on chewiness and the psyllium's reduced impact on moderate contribution, it is imperative to optimize the formulation in order to achieve a less chewy structure in gluten‐free cakes.

### 3.8 Resilience

Resilience is defined as a product's ability to “fight back to its original height” (Lu et al., 2010). In the current investigation, the resilience of the gluten‐free cupcakes altered to 0.285 and 0.375 (Table 3). The lowest resilience value was recorded while using the air fryer method with 1.2% stevia and 0% psyllium and the highest resilience was achieved with the oven method using 0% stevia and 4% psyllium. Based on the ANOVA data, the linear model is significant, with an *F*‐value of 19.67 and *p*‐values <0.0001 (Table 5). In the current investigation, only the linear components (*A*, *B*, and *C*) were statistically significant (*p* ≤ 0.05). Lowering *p*‐values and a proportionally increased sum of squares indicate the significance of the independent variables on the response. The significance of variables is prioritized as follows: baking type (*C*), stevia (*A*), and psyllium (*B*). The *R* ^2^, adjusted *R* ^2^, and CV values were 0.72%, 0.69%, and 3.15%, respectively. The polynomial Equation (11) of the linear model for resilience represents the interaction of the independent variables (*A*: stevia, *B*: psyllium, *C*: baking type) on the relevant response (*Y*_8_).

(11) $$Y_{8}=0.33492-0.012\times A+0.012\times B+0.0104231\times C$$

Figure 2d,h shows graphical representations of regression Equation (11), which are used to investigate the combined effect of stevia, psyllium, and baking types on resilience. Both figures clearly demonstrate that an increase in psyllium progressively enhanced resilience, whereas an increase in stevia had a slightly decreasing influence on the response. These results are somehow in agreement with the observations of Gao et al. (2017), who reported that increased stevia concentration correlates with denser, less elastic, and less resilient cakes. However, in Figure 2h, oven baking showed greater overall resilience than air fryer baking.

In a study conducted by Filipčev et al. (2021), gluten‐free bread was enriched with psyllium, xanthan gum, guar gum, and HPMC. The results indicated that psyllium exhibits positive textural properties, specifically the lowest crumb hardness and the highest crumb resilience. This was associated with the water‐holding capacity of psyllium fibers and its interaction with proteins, which were all influencing the texture and strength of the dough. The cohesiveness and resilience of crumb were more dependent on starch gelatinization and swelling than on gluten aggregation, as claimed by Renzetti and van der Sman (2022).

### 3.9 Pore characteristics

The pore characteristics of the gluten‐free sugar‐reduced cake were investigated in the present research by employing a calculation of pore number. The results of ANOVA shown in Table 5 indicate that the proposed model is significant with an *F*‐value of 6.25 and a *p*‐value of 0.001. The regression equation's lowest *p*‐value and maximum magnitude of the linear terms of psyllium (*B*), which had the most profound impact on the pore numbers. The linear term of the baking type, the quadratic term of *B* ^2^, and the interaction terms of *ABC* and *B* ^2^ *C* come next. The model's reliability is reinforced by the following *R* ^2^, adjusted *R* ^2^, and CV values: 0.83%, 0.70%, and 19.59%, respectively. The Equation (12) indicates that only the linear terms of *B* and *C* possess positive values. Only *B* and *C* terms had a growing effect on response, as positive magnitude indicates an increase in relevant response as the independent variables increased. In terms of coding variables, Equation (12) showed the association between the number of pores (*Y*_9_) and the independent variables psyllium (*B*), baking style (*C*), and stevia (*A*).

(12) $$\begin{array}{ccc} Y_{9} & = & 34.53-1.33\times A+9\times B+8.32\times C-1.5\times AB\\ & & -\;0.66\times AC+2.5\times BC+4.37\times A^{2}-8.62\times B^{2}\\ & & -\;6.25\times ABC+0.10\times A^{2}C-8.39\times B^{2}\;C \end{array}$$

**FIGURE 3:**
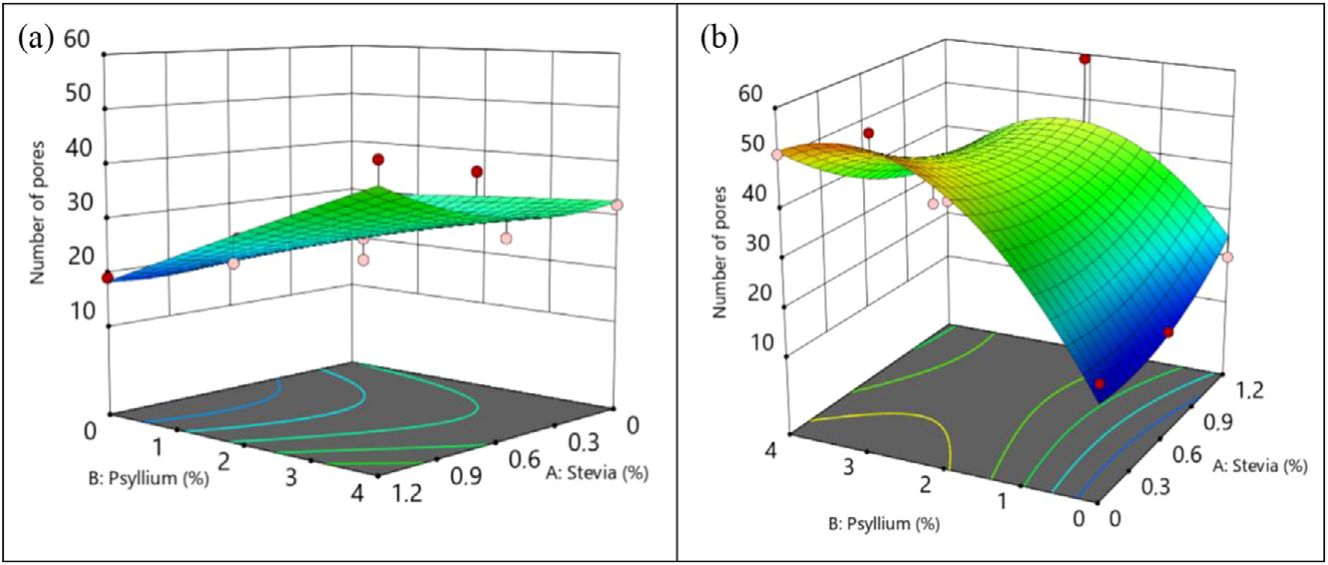
Pore numbers of response surface 3D plots for the interaction factors of stevia, psyllium, and the type of baking (left column: air fryer, right column: oven, red dots: experimental runs.).

**FIGURE 4:**
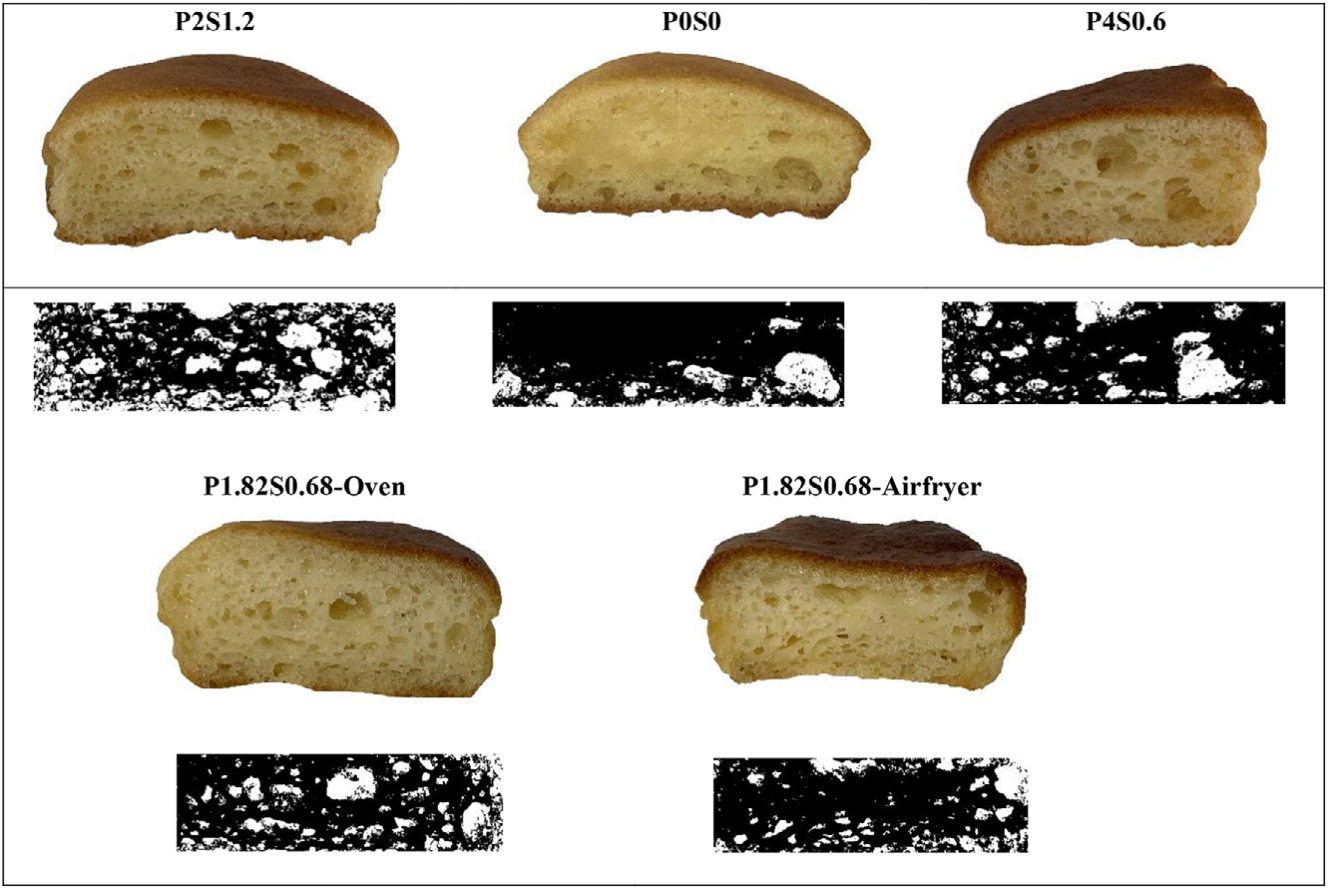
Representative pictures and binary images of cross‐sectional area of cakes (P2S1.2: addition of psyllium 2%, stevia 1.2% at oven; POSO: addition of psyllium 0%, stevia 0% at oven; P4S0.6: addition of psyllium 4%, stevia 0.6% at airfryer; P1.82S0.68 (produced at optimum formulation: addition of psyllium 1.82%, addition of stevia 0.68%).

The graphs in Figure 3 depicted how the inclusion of stevia, psyllium, and the baking process affects the number of pores in the finished cupcakes. Additionally, Figure 4 illustrates the cross‐section of the cakes and their binary images. The data presented in Table 3 indicate that the cupcake pore counts vary from 15 to 59. The lowest number of pores was obtained in the control cake with 0% stevia and 0% psyllium baking in the oven (Figure 4, P0S0) and the cakes with the maximum number of pores were produced at 1.2% stevia and 2% psyllium in the oven (Figure 4, P2S1.2). The pores were generally more uniformly distributed and displayed higher homogeneity and aerated in the oven than in the air fryer, particularly at higher concentrations of psyllium (Figure 3a,b). According to Ngan et al. (2023), despite the relatively significant volume expansion of the cupcakes in the air fryer, there was less homogeneity and random bumps on the upper surface of the cake. This is explained by the forced heated air flow that resulted in a less porous and denser crumb structure. Our study supports these observations. Unequal pore size and nonuniform pore distribution of the cake were noticed in air fryer baking method as shown in Figure 4 (P4S0.6). Air bubbles of a smaller dimension are more advantageous for the production of cakes with a suitable texture and volume (Yıldız et al., 2018). Also, the appearance of bigger holes could be explained by the puffing effect caused by the increased pressure gradient inside the cakes (Ozkahraman et al., 2016).

As shown in Figure 3a, when cakes are baked in the air fryer, increasing psyllium concentration gradually increased the pore numbers in cupcakes. However, until 0.6% stevia concentration, the plot exhibited a reasonably flat trend. After that, increasing the stevia level increased pore growth in the air fryer. Psyllium has a more noticeable effect on the number of pores when the cakes were baked in the oven, as shown in Figure 3b. There is a substantial increase in pore numbers as the concentration of psyllium increases. However, beyond a certain concentration, the number of pores reaches a plateau. According to the aforementioned studies, adding hydrocolloid to baked products significantly enhanced the volume, porosity, softness, and capacity of absorbing water (Salehi, 2019). Several gluten‐free formulations have been modified to enhance the sensorial/textural properties and dough performance of the gluten‐free product by incorporating xanthan gum, guar gum, carrageenan, locust bean gum, or pectin (Salehi, 2020). The xanthan and xanthan–guar gum combination results in the gluten‐free rice cakes with the highest number of pores (Turabi et al., 2010). In another study, the viscoelasticity of gluten‐free cake batter was enhanced by adjusting the proportions of maize flour, rice flour, olive pomace, and xanthan gum (Balbinot Filho et al., 2022). The addition of 0.25% xanthan gum entrapped air bubbles and provided a more homogeneous structure than the comparable formulations without xanthan gum. According to Majzoobi et al. (2017), a mixture of 1.5% xanthan and 1.5% pectin resulted in a satisfactory gluten‐free cake that was then augmented with carrot pomace. Similar to this, psyllium fiber and baking technique had significant effects on the bread's formation of more even and fine pores (Wojciechowicz‐Budzisz et al., 2023). Plant‐based fibers or sugar alcohols are reported to increase the temperature of starch gelatinization and alter the volume of baked goods. This implies that, although psyllium supports pore formation to a certain extent, an excessive quantity resulted in a denser batter that restricts further expansion, leading to a flattened attitude in the number of pores. It is evident that optimizing psyllium levels is crucial for achieving the desired textural properties.

Furthermore, according to Figure 3b, the effect of stevia was limited when compared to psyllium in the oven baking method. However, when the stevia concentration exceeded 0.6%, a small increase in the number of pores was found. It was concluded that the use of stevia at higher levels in both methods can result in a more porous structure in cakes. According to past studies, 100% sugar replacement led to undesirable characteristics in terms of aeration, final volume, pore numbers, and pore expansion. Nonetheless, combining sweeteners (stevia, inulin, and bovine plasma protein) with sucrose increased the percentage of aeration and volume in muffins (López et al., 2022).

### 3.10 Optimization

The best combination of stevia, psyllium, and baking type for gluten‐free sugar‐reduced cupcake formulation was identified by maximizing the response outcomes of specific volume, springiness, and number of pores while minimizing hardness, cohesiveness, gumminess, chewiness, and resilience. The optimum cake formulation, determined by numerical optimization, was predicted to consist of 0.68% stevia and 1.82% psyllium, employing the oven method. Under these optimized circumstances, responses are expected to have 35.88 specific volume, 1731.96 hardness, 0.937 springiness, 0.657 cohesiveness, 1141.76 gumminess, 1068.81 chewiness, 0.343 resilience, and 41.65 pore numbers. The desirability approach is a well‐established tool for optimizing design variables, including single and multiple responses, with values ranging from 0 to 1 (Kalkan & Maskan, 2024). In the current investigation, the desirability of the cake was found to be 0.7. In order to verify the results, the optimized cake was produced at the predicted condition (0.68% stevia, 1.82% psyllium in the oven) and yielded the following results: 38.75 (mL/g) specific volume, 1710.85 hardness, 0.950 springiness, 0.638 cohesiveness, 1119 gumminess, 1042 chewiness, 0.350 resilience, and 44 pore numbers. When the optimized‐oven cupcake was compared to the control, it revealed that the optimized‐oven‐baked cupcake was superior in terms of specific volume, all textural qualities, and pore numbers. Therefore, binary images of optimized cupcakes that were produced using both baking methods were displayed in Figure 4‐P1.8S0.6‐Oven and P1.8S0.6‐Airfryer. The predicted and experimental values were found to be in close alignment at the optimized formulation. The results indicate that the regression model worked well at anticipating the optimized formulation of gluten‐free, sugar‐reduced cake.

### 3.11 Sensory properties

Sensory science provides objective information about the consumer understanding of a product. Sensory analysis examines the properties (texture, flavor, taste, appearance, smell, etc.) of a product or food through the senses (sight, smell, taste, touch, and hearing) of the panelists. This type of analysis has been used for centuries for the purpose of accepting or rejecting food products.

**FIGURE 5:**
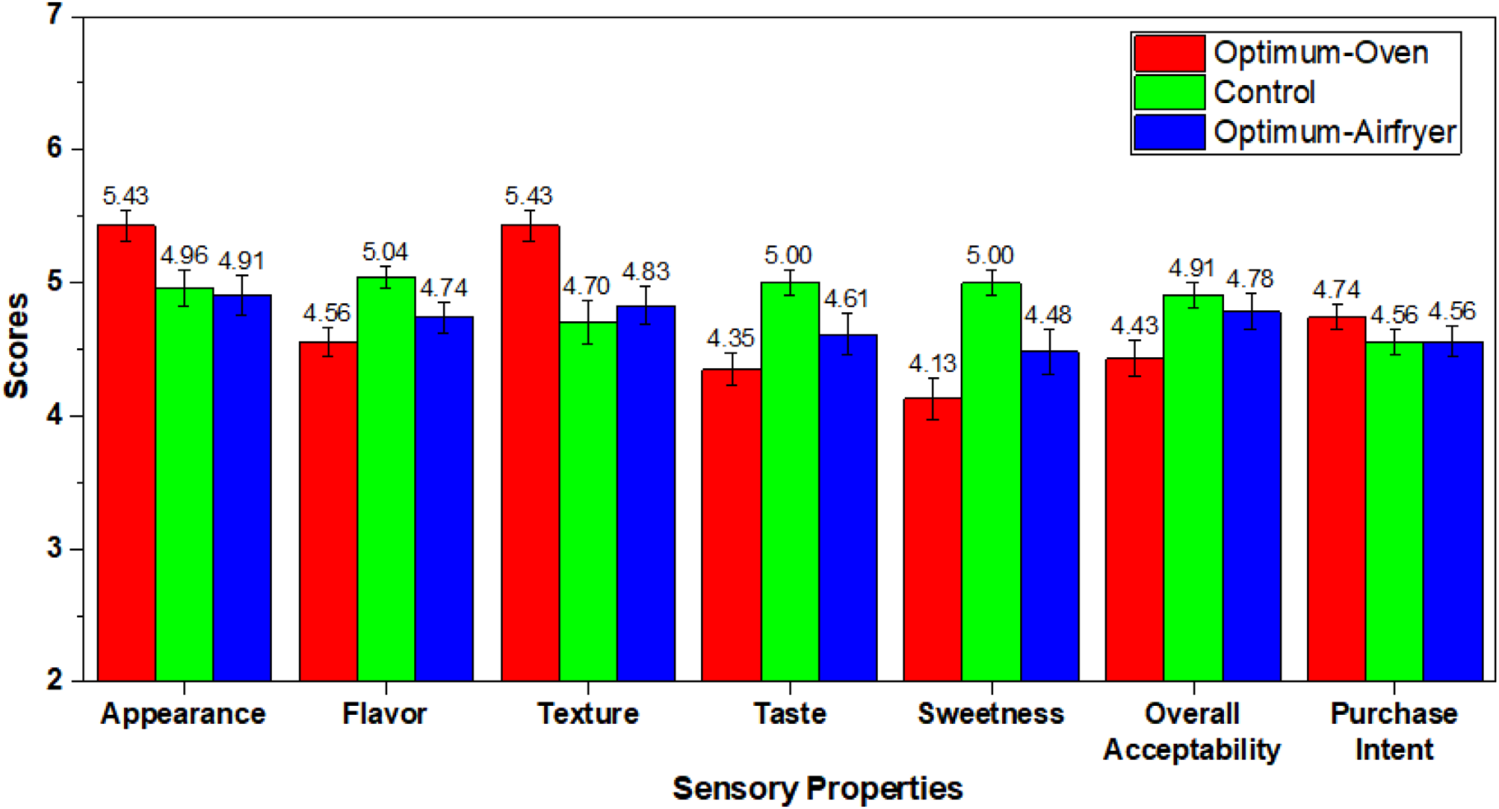
Sensory results of gluten‐free control and cupcakes produced at optimum formulation.

Sensory analysis of three cupcake samples (produced in oven at optimum conditions, produced in air fryer at optimum conditions, and control) was performed. The results of sensory evaluation of gluten‐free sugar‐reduced cupcakes, optimized formulation, and control were scored in terms of several attributes as illustrated in Figure 5. In terms of appearance and texture, cupcake with optimum formulation and baked in oven received the significantly highest score (5.43), while no significant differences were found between optimized air fryer and control in both attributes. In answer to consumer demand for softer textures and less hardness in cakes, the optimized formulation (1.82% psyllium and 0.68% stevia) and oven‐baking method contributed to a remarkably higher texture score (5.3) for the optimized oven‐baked cupcake. The optimized oven‐baked cake's sensory and texture properties matched since oven‐baked cakes are generally lower in hardness. The optimized air fryer and control did not exhibit any significant differences in either of these attributes. This can be related to the favorable impact of psyllium and the oven baking technique on gluten‐free cupcakes. The incorporation of psyllium at 1.82% and stevia at 0.68% enhanced the willingness and attractiveness of the cakes when baked in an oven, in comparison to those baked in an air fryer and the control group. To be considered acceptable, a product's sensorial properties must have an acceptability index of at least 70%, according to Ávila et al. (2017). The acceptability index for the improved cupcake was determined to be 77% for appearance and texture.

On the other hand, the control cupcake received the highest rating in terms of overall acceptability, sweetness, flavor, and taste. Also, the optimized‐air fryer cupcake was the second most acceptable sample in terms of these qualities. Sensory analysis revealed that the optimized cupcakes were generally well‐received and should be improved as reflected by the AI, which ranged from 59% to 77%. The acceptance of flavor ranging from 4.56 to 5.04, taste from 4.35 to 5.00, sweetness from 4.13 to 5.00, and overall acceptability ranging from 4.43 to 4.91, as represented on the hedonic scale by “neither like nor dislike to I like very much.” According to Ngan et al. (2023), green tea‐incorporated cupcakes offered better taste in oven baking than air fryer baking method. In another investigation, there was no noticeable difference found between oven‐baked and airfryer‐baked cakes for overall acceptability, aroma, and sweetness (Mior Zakuan Azmi et al., 2019).

Stevia offers a refreshing, sweet taste that lasts for hours in the mouth (Amarakoon, 2021). Even though the batter was the same, the optimized‐air fryer cupcake scored higher for flavor, sweetness, and overall acceptability than the optimized‐oven cupcakes. Panelists generally reported that the metallic flavor of the cake was less noticeable when baked in an air fryer as opposed to an oven. It could be linked to the oven‐baked cupcakes' significant moisture retention during baking, while stevia served as a humectant and diffused more effectively in the oven during baking. As a result, the sensation of metallic flavor and bitter taste is less effective in an air fryer than in an oven.

In the same vein, juices and dairy products were reported to have a bitter taste and a disagreeable metallic flavor due to stevia (Yildiz & Karhan, 2021). The consumer's purchase intention test yielded a score of 4.56 for the control and optimized airfryer and 4.74 for the optimized oven cupcake, as a result of the positive values associated with sensorial evaluation shown in Figure 5. The expression “probably would by the product” corresponds to these values.

## 4 CONCLUSION

The current study successfully developed a new formulation of gluten‐free, sugar‐reduced cupcakes by assessing the effects of stevia, psyllium, and two different baking types (oven and air fryer). The effect of different baking methods on the developed cakes was clearly observed in runs 9 and 23, primarily due to the absence of stevia and psyllium. Air‐fried cakes demonstrated higher values than oven‐baked cakes in terms of baking loss (9.77% and 9.55%, respectively), specific volume (22.46 and 20.85 mL/g, respectively), hardness (3011.45 and 2040.32 gf, respectively), gumminess (1904.87 and 1319.45, respectively), and chewiness (1764.74 and 1222.83, respectively). Meanwhile, for cohesiveness (0.648 and 0.633, respectively) and resilience (0.3480 and 0.3245, respectively), oven‐baked cakes gave higher results than air‐fried cakes. For springiness, no significant difference was observed in cakes (0.927 and 0.926, air fryer and oven, respectively). According to Mior Zakuan Azmi et al. (2019), the similar result was reported for springiness for both baking methods. The findings of this study support the prior research (Al‐Muhtaseb et al., 2013; Mior Zakuan Azmi et al., 2019; Ngan et al., 2023) and are of important value to the literature, as they address a significant gap in existing knowledge. The strength of this study lies in being the first to combine 1.82% psyllium and 0.68% stevia in a 60% sugar‐reduced batter, generating a gluten‐free cake that had superior characteristics than the control in terms of specific volume, hardness, springiness, cohesiveness, gumminess, chewiness, resilience, number of pores, and sensory properties of appearance and texture. Moreover, it is noteworthy that, apart from the sensory properties of appearance and texture, the optimized‐airfryer cake was rated as the second‐best option by the panelists. The number of pores increased by 2.5 times, from 17 to 44, in the optimized‐oven cupcakes compared to the control, demonstrating the profound effect of ingredient optimization and baking type. This study identified a promising alternative for individuals suffering from chronic disorders, particularly celiac disease, gluten intolerance, Type 2 diabetes, and obesity, who are seeking “clean label” products for health concerns. The combination of stevia as a natural sweetener and psyllium as a gluten substitute enhances the qualities of low‐calorie, gluten‐free cupcakes. As indicated by the purchase intention test (score of 4.74 over 7), consumers preferred the optimized‐oven‐baked cupcakes.

## AUTHOR CONTRIBUTIONS

**Ezgi Kalkan**: Conceptualization, data curation, formal analysis, investigation, methodology, project administration, software, validation, visualization and roles/writing—original draft. **Medeni Maskan**: Conceptualization, data curation, investigation, methodology, project administration, supervision, validation, visualization and writing—review and editing.

## CONFLICT OF INTEREST STATEMENT

The authors declare no conflicts of interest.

## ETHICS STATEMENT

All experimental procedures were performed in accordance with a protocol approved by The Ethics Committee of Gaziantep University in Turkey (protocol number 012, record date: 17.09.2024).
